# Cross-border malaria in the triple border region between Brazil, Venezuela and Guyana

**DOI:** 10.1038/s41598-022-05205-y

**Published:** 2022-01-24

**Authors:** Rispah Abdallah, Jaime Louzada, Christina Carlson, Dragan Ljolje, Venkatachalam Udhayakumar, Joseli Oliveira-Ferreira, Naomi W. Lucchi

**Affiliations:** 1 Malaria Branch, Division of Parasitic Diseases and Malaria, Center for Global Health, Centers for Disease Control and Prevention, Atlanta, USA; 2 Oak Ridge Institute for Science and Education (ORISE), Center for Global Health, Centers for Disease Control and Prevention, Atlanta, USA; 3 Federal University of Roraima, Roraima, Brazil; 4 Williams Consulting LLC, Catonsville, USA; 5 Institute Oswaldo Cruz-Fiocruz, Rio de Janeiro, Brazil

**Keywords:** Malaria, Epidemiology

## Abstract

The state of Roraima, in Brazil, has recently seen an increase in the number of reported *Plasmodium falciparum* infections believed to be imported from neighboring countries. The objective of this study was to determine the prevalence of *Plasmodium* species among patients attending malaria health posts in Roraima and quantify the infections attributable to imported malaria. This cross-sectional case study was carried out between March 2016 and September 2018. Study participants were recruited as they exited the malaria health post. Information about residence, occupation and travel history was collected using a questionnaire. A dried blood spot was collected and used for malaria diagnosis by PCR. A total of 1222 patients were enrolled. Of the 80% *Plasmodium* positive samples, 50% were *P. falciparum*, 34% *P. vivax*, 8% mixed *P. falciparum/P. vivax* and 0.2% mixed *P. falciparum/P. ovale* infections and 8% tested positive for *Plasmodium,* but the species could not be identified. 80% of the malaria patients likely acquired infections in Venezuela and the remaining 20% acquired in Guyana, Brazil, Suriname and French Guyana. 50% of the study participants reported to be working in a mine. Results from this study support the hypothesis that imported malaria contribute to the bulk of malaria diagnosed in Roraima. These findings are in keeping with previous findings and should be considered when developing malaria control interventions.

## Introduction

Malaria continues to be a major global public health problem with about 229 million new malaria cases and approximately 409,000 deaths worldwide reported in 2019^1^. The incidence rate of malaria was estimated to have decreased by 18% globally between 2010 and 2016, but despite this reduction, a substantial increase in cases was recorded in the Americas between 2014 and 2015, mainly due to increases in cases in Brazil and the Bolivarian Republic of Venezuela (hereinafter referred to as Venezuela)^2^. In Brazil, the number of malaria cases, rose from 143,748 in 2015 to 193,837 in 2018 after seven years of decline and in Venezuela increased from 91,918 in 2015 to 242,561 in 2016^3^. The number of malaria cases in Venezuela reached 414,527 in 2017, mainly in the Bolivar state in the northeast of the country^3,4^.

Malaria transmission in Guyana, remains concentrated in mining populations in the Amazonian Regions 1, 7, 8 and 9 with these regions accounting for 85–95% of the total malaria cases observed in the country^5^. These four regions border Venezuela (regions 1 and 7) and Brazil (regions 8 and 9). According to the World Malaria Report, 11% of Guyana’s population of about 746,955 people lives in these malaria high transmission areas^6^. Malaria cases in Guyana decreased by 68.3% between 2013 and 2015 (21,495 cases), however, case incidence of more than 40% was reported in 2020 compared to 2015. Historically, Guyana has been a hotspot for illegal gold-mining, an activity that has been associated with human migration and malaria transmission in the Amazon and more worrisome, artemisinin resistance is suspected to be emerging in Guyana^7^.

**Figure 1:**
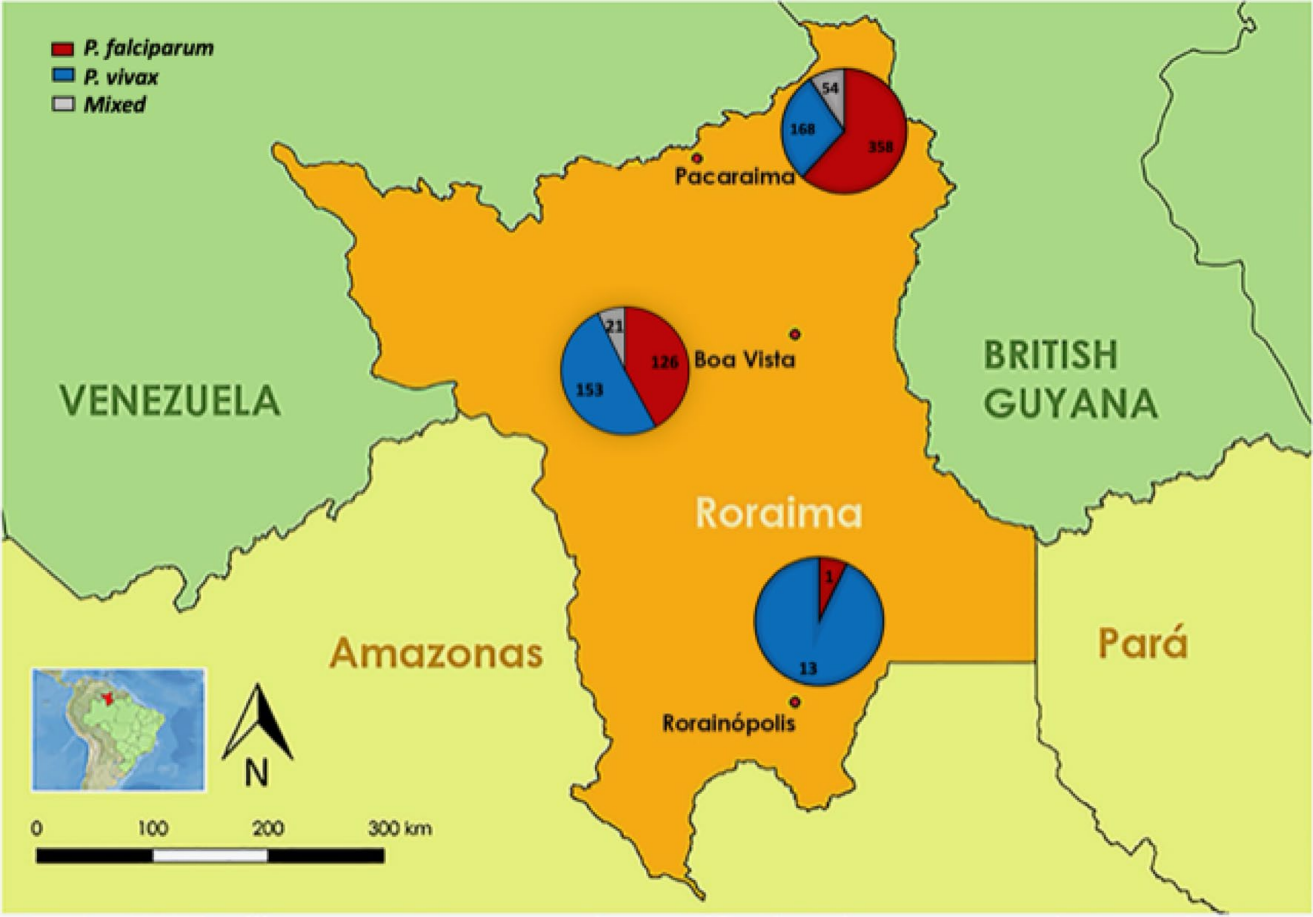
A map of Roraima state and the distribution of malaria cases by species in the municipalities of Pacaraima, Boa Vista and Rorainópolis. The study was carried out in three municipalities in Roraima state: Pacaraima, Boa Vista and Rorainópolis. The distribution of *Plasmodium* species in the three study sites is shown using different pie-charts for each site. Mixed infections were only observed in Boa vista (21 *P. falciparum/P. vivax*) and Pacaraima (53 *P. falciparum/P. vivax* and 2 *P. falciparum/P. ovale*). The map was created in Quantum GIS software (https://www.qgis.org, QGIS version 2.14.17), using shapefile layers from South America and the state of Roraima. The coordinates of the municipalities of Boa Vista, Pacaraima and Rorainópolis was added to the map in addition to pie charts built in the Microsoft Excel software.

Roraima is the Brazilian state in the extreme north of the country that shares borders with Guyana and Venezuela (Fig. 1). It is part of what is referred to as the Guiana Shield consisting of Suriname, Guyana and French Guiana and some parts of Venezuela, Colombia and Brazil. Roraima is considered to have a moderate risk for malaria transmission. However, the state has recently seen an increase in the number of reported malaria cases from 5713 in 2014 to 23,369 in 2018, especially an increase in *P. falciparum* infections responsible for 26% of the imported cases^3,8^. Since 2016, Roraima has experienced the impact of an unprecedented migratory flow of Venezuelans due to the economic and political crisis in their country^3^. According to the Brazilian Ministry of Health, the cases imported from Venezuela also increased from 2470 cases in 2016 to 4478 cases in 2018 of which 1247 were *P. falciparum*. Exacerbating the situation in this region is the presence of the gold mining activities with poor working and living conditions and migration back and forth from one country to the other, all of which have been shown to cause an increase in malaria cases and spill over to neighboring countries^3^. In 2018, gold from 862 illegal mines was the second most exported product from Roraima^9^.

The proximity of Roraima state to Guyana and Venezuela and the presence of highly mobile populations in this region due to the presence of many mining activities contribute to the importation of malaria for this state^9,10^. A recent publication reported that both Venezuela and Guyana contributed to the majority of cross-border cases into Roraima from 2012 to 2018^11^. The objective of this study was to determine the prevalence of *Plasmodium* species among malaria patients attending health clinics in Boa Vista, Pacaraima and Rorainópolis and quantify the infections attributable to imported malaria.

## Results

A total of 1222 patients were enrolled between March 2016 and September 2018. A summary of the study’s participants is shown in Table 1. Majority of the study participants were enrolled in Pacaraima (53%) and Boa Vista (42%) with only 5% enrollment in Rorainópolis. Over 70% of the participants were males and the mean age was 37 years. A total of 1217 participants provided occupation/profession information and half of these (50%) reported to be miners or working in a mine (Table 1) Miners were seen only in Boa Vista and Pacaraima and none in Rorainópolis. A large proportion of participants (47.3%) reported living outside of Brazil mostly because of their job. This was especially evident in Pacaraima where 89% of the patients reported living in Venezuela, in contrast to Rorainópolis and Boa Vista where 100% and 99.4% respectively, reported living in Brazil (Table 1).

**Table 1:** Characteristics of study participants (n = 1222).

	Boa Vista	Pacaraima	Rorainópolis	Overall
	n = 520	n = 645	n = 57	n = 1222
Age in years, mean (range)	36 (17–64)	36 (09–89)	38 (15–70)	37
**Gender**				
Male, n (%)	388 (75%)	444 (69%)	32 (56%)	864 (71%)
**Occupation**^**a**^				
Mining, n (%)	342 (66%)	269 (42%)	0 (0%)	611 (50%)
Others,^b^ n (%)	177 (34%)	373 (58%)	56 (100%)	606 (50%)
**Country of residence**				
Brazil, n (%)	517 (99.4%)	70 (10.9%)	57 (100%)	644 (52.7%)
Venezuela, n	3 (0.6%)	575 (89.1%)	0 (0%)	578 (47.3%

^a^Occupation reported for 1217 participants (missing 1 from Boa Vista, 3 from Pacaraima and 1 from Rorainópolis).

^b^Others: students, lawyers, housewives, drivers, cooks, salesmen, working in agriculture sector, and merchants.

### Malaria cases observed

Of the 1222 samples collected, PET-PCR identified 983 (80.4%) *Plasmodium* positive cases, of which 334 (34%) were *P. vivax*, 491 (50%) *P. falciparum*, 74 (8%) mixed *P. falciparum/P. vivax* and 2 (0.2%) mixed *P. falciparum/P. ovale* infections. A total of 82 (8%) samples tested positive for *Plasmodium,* but the species could not be identified. The PCR Ct value of the 82 samples where only the *Plasmodium* genus but not the species could be detected ranged from 33.72 to 39.86 suggesting low parasite density infections.

### Probable origin of malaria infection

**Figure 2:**
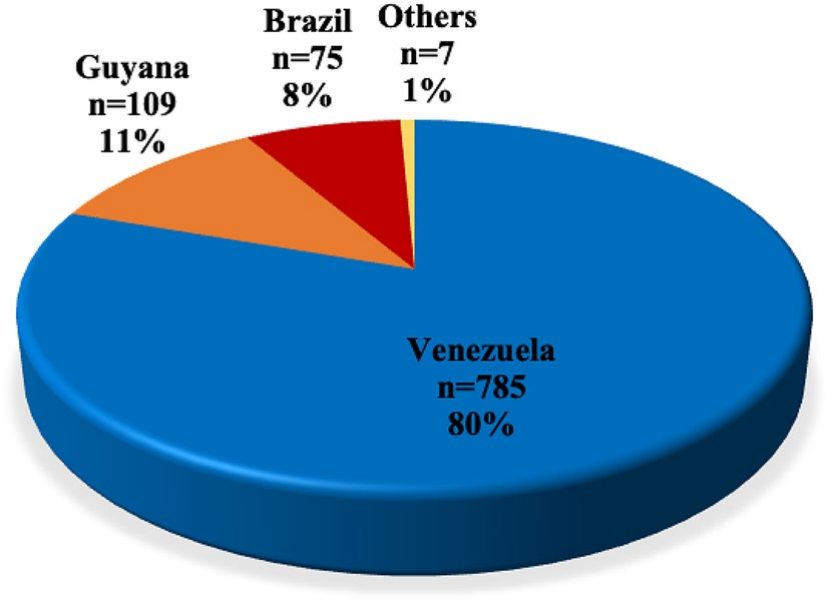
Number and frequency of malaria cases recorded in the municipalities of Pacaraima, Boa Vista and Rorainópolis, Roraima state, according to the probable origin of malaria infection. The probable county of origin of malaria infections based on the study participant’s reported country of residence and travel history is shown. The majority of infections were acquired in Venezuela, followed by Guyana and Brazil. Others are infections acquired in Suriname (2) and French Guiana (5).

**Figure 3:**
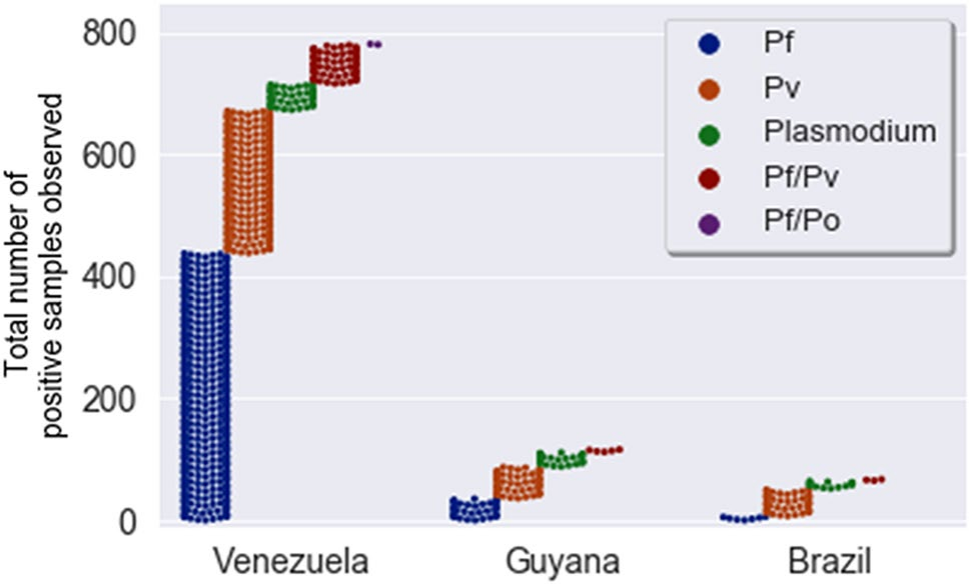
Number of malaria positives samples stratified by *Plasmodium* species and the probable country of origin of infection. The total number of malaria isolates observed in the three main countries of probable origin of infection (Venezuela, Guyana and Brazil) is shown. The different colors represent different species with each dot representing a sample. A total of 82 samples tested positive for *Plasmodium,* but the species could not be identified. Pf = *P. falciparum*, Pv = *P. vivax*, Po = *P. ovale.* Figure does not include 3 samples from Suriname (1 (Pf; 1Pv; and 1Pf/Pv) and 2 from French Guiana (Pf).

Of the 983 positive cases, 976 provided travel and domicile information. The provided information was used to determine the most probable country of origin of the malaria infection. The majority of the malaria cases observed in our study were imported from Venezuela (80%), Guyana (12%), Suriname (0.5%) and French Guiana (0.2%). Only 7% of these were locally acquired in Brazil (Fig. 2). No differences were observed in the age, gender and occupation of the patients with locally acquired infections compared to imported cases (p > 0.05) except for the fact that the majority of infections acquired in Brazil (autochthonous infections) were *P. vivax* in contrast to those acquired from Venezuela, the majority of which were *P. falciparum* infections (Fig. 3, Table 2).

**Table 2:** Distribution of autochthonous and imported cases by *Plasmodium* species and study site.

	Malaria diagnosis	Autochthonous, n (%)	Imported, n (%)
Boa Vista, N = 375	*P. falciparum*	3 (2.4)	123 (97.6)
	*P. vivax*	27 (17.6)	126(82.4)
	Mixed (Pf + Pv)	4 (19.0)	17 (81.0)
	*Plasmodium*	13 (17.3)	62(82.7)
	Total	47 (12.5)	328 (87.5)
Pacaraima, N = 586^a^	*P. falciparum*	6 (1,7)	352(98.3)
	*P. vivax*	8 (4.7)	160 (95.3)
	Mixed (Pf + Pv/Pf + Po)	0	54 (100)
	*Plasmodium*	0	6 (100)
	Total	14 (2.4)	572 (97.6)
Rorainópolis, N = 15	*P. falciparum*	0	1 (100)
	*P. vivax*	13 (100)	0
	Mixed	0	0
	*Plasmodium*	1 (100)	0
	Total	14 (93.3)	1 (6.7)

^a^Travel and country of origin of infection not provided for 7 samples (6 with *P. falciparum* and 1 Pf + Pv).

### Malaria species stratified by site

Of the malaria cases recorded in our study, majority were observed in Pacaraima, 593/983 (60%), followed by Boa vista, 375/983 (38%) with Rorainópolis having the least number of malaria cases, 15/983 (2%) (Table 2). Over 85% of the malaria cases observed in Pacaraima and Boa Vista were imported in contrast to what was observed in Rorainópolis (Table 2). Not surprisingly, the proportions of *P. falciparum* and *P. vivax* infections were found to differ by study site, X^2^ (2, N = 825) = 57.30, p < 0.001; the majority (62%) of infections in Pacaraima were caused by *P. falciparum*, in contrast to Rorainópolis where 93% of the infections were caused by *P. vivax*. Boa Vista had an almost equal distribution of the two species (Fig. 1 and Table 2).

## Discussion

Results from our study showed that the majority of malaria cases observed in Boa vista and Pacaraima, during our study period (2016–2018) were imported from the neighboring countries majority of which were from Venezuela and cases observed in Rorainópolis (2018), despite the lower sample size, were mainly autochthonous. This supports previous observations in Roraima^12–14^. The border municipality of Pacaraima and the capital Boa Vista are the municipalities with the highest number of imported cases of malaria as many patients cross the border in search of treatment since malaria diagnosis and treatment in Brazil is free to all people in the national territory, including foreigners whether students, tourists or refugees, with or without a visa. The influx of patients from Venezuela has been attributed to the political conflicts in the country and migration to Brazil in search of employment in addition to treatment. This surge in malaria cases can strain the health care systems that typically cater to a smaller number of cases and can lead to an increase in autochthonous malaria infections^9,14,15^. For example, both Boa Vista and Pacaraima municipalities have a low risk of malaria transmission, however, as per the Brazilian Ministry of Health, malaria cases have been gradually increasing in these municipalities and between 2016 and 2018, 6368 cases were recorded, of which 1958 (31%) were autochthonous and 4410 (69%) imported, underlining the complexity of malaria control in this this region^8^. In contrast, imported malaria in Rorainópolis, in the same period was low, 622 (8%) compared to 6767 (92%) out of a total of 7389 malaria cases registered in that municipality^8^. This is not surprising given that Rorainópolis is a malaria endemic municipality that borders the state of Amazonas and three other high malaria municipalities thus favoring the maintenance of local transmission in this region^9^.

In general, the predominant *Plasmodium* species reported in Brazil is *P. vivax,* contributing to about 77% of the infection^15,16^. In keeping with this trend, the majority of the autochthonous cases observed in our study were *P. vivax* infections (mainly in Rorainópolis) while *P. falciparum* infections were contributed largely by patients from Venezuela and Guyana observed in Boa Vista and Pacaraima. Recent studies have observed similar trends of higher reports of *P. falciparum* cases reported with the influx of people from Venezuela^2,14^. *P. falciparum* is more commonly associated with severe disease manifestations than *P. vivax* and is treated differently. In Brazil, artemether-lumefantrine is used for the treatment of *P. falciparum* while *P. vivax* is treated with chloroquine (if chloroquine resistance does not exist) or artemether-lumefantrine plus a radical cure (to get rid of hypnozoites) with primaquine. Therefore, an increase in *P. falciparum* infections in a country that largely has *P. vivax* infections has implications for the malaria control program and need to be considered while making policies for malaria control in the region.

Over half of the participants in our study self-reported their occupation as miners or as working in mines in Brazil or the neighboring countries (Venezuela, Suriname, French Guiana or Guyana). The impact of imported malaria by gold miners in Roraima was previously reported^9^. Brazilian miners in Roraima spend part of their time between the gold mines abroad and Boa Vista, where they live and sell the products of this labor, generating high mobility between endemic areas in Venezuela and Guyana to Boa Vista^9^. The incidence and prevalence of malaria in mining cities or camps has been shown to be high^3,10,17,18^. This is because the environmental and social-economic situations in mining cities and camps makes for fertile grounds for malaria transmission: mining activities often lead to deforestation and development of stagnant water which favor the breeding of mosquitos (malaria parasite vectors); the workers are exposed to mosquito bites working outdoors for long periods of times and sleeping in makeshift accommodation; this also results in a highly mobile group of young people who migrate from country to country in search of jobs and often many of the mining camps lack good health care and disease prevention and control measures. The movement of mine workers from mine to mine and back to visit their families has led to spikes in malaria cases and outbreaks in bordering countries^14^. For example, a recent study reported on the resurgence of malaria in the Amazonian border area between French Guiana and Brazil^19^. Indeed, the transmission of malaria in the Guiana Shield has fluctuated over the years due to the gold mining and the constant migration of people within the region.

In addition, there is a potential risk of spread of artemisinin resistant *P. falciparum* parasites as Guyana has reported parasites with *kelch 13* C580Y mutations associated with artemisinin resistance^20,21^. This observation along with the high risk behavior of miners in terms of using inappropriate self-medication among miners and challenges to appropriate malaria treatment in Venezuela due to political crisis and their high migration rate adds further fuel to potential spread of artemisinin parasites in this region^10,22,23^.

While majority of the patients seen in both Boa Vista and Pacaraima return to their workplace (often mines in neighboring countries) after treatment, the high numbers of imported malaria cases can eventually lead to high local malaria transmission over time especially if the right mosquito vectors are in circulation. Therefore, understanding the Anopheles species in circulation in both Boa Vista and Pacaraima is important. Little is known about the Anopheles species in circulation in Pacaraima, but some studies have been carried out in Boa Vista where *An. darlingi* and *An. albitarsis s.l.* were incriminated as the main malaria vector^24^. *An. darlingi* is a very competent and the main malaria parasite vector in Brazil while *An. albitarsis* s.l seem to participate in malaria transmission, usually as secondary vectors because they are unable to sustain disease transmission in the absence of the *An darlingi *^25^. Studies are in progress by our group to evaluate anopheline species habitats, behavior, distribution, and seasonal occurrence in Boa Vista and Pacaraima. This surveillance of Anopheles in the studied sites is an essential component in malaria control strategies and continuous surveillance of imported malaria cases and Anopheles larval sites and the association of these surveillances with additional malaria control efforts such as spraying of households and health education specially among the miners is necessary.

Some limitations of our study include the fact that classification of imported cases was based on self-reported travel history. The use of parasite genotyping using approaches such as microsatellites markers would have provided additional support that cases were indeed imported however, this was not performed in this study. Secondly, the use of a survey questionnaire, while utilized in many studies, has limitations in that participants might not always tell the truth. This could have affected the accuracy of information we collected such as the origin of infection and patient occupation. However, while this is a valid point, it is also a difficult one to prove. Finally, participants in our study were enrolled as they exited a malaria health post, typically visited by symptomatic patients seeking a malaria diagnosis and treatment. This biased toward malaria positive participants which explains the high test positivity we observed that is not generalizable given the sampling we used. However, according to the Ministry of Health the prevalence of malaria positive individuals and imported cases reported at health post where we collected samples was also high. For example, in 2016, the malaria post in Pacaraima examined 1772 thick blood smears and 48% (n = 846) were positive and 99% (n = 839) were imported from Venezuela. Likewise, in Boa Vista the heath post examined 3833 blood slides and 31% (n = 1173) were positive and 99.8% (n = 1171) were imported. In Rorainópolis, an endemic area for malaria and the second largest city in the state, 3832 thick blood slides were examined and 11.5% (n = 442) were positive for malaria and 86% (n = 380) of these were autochthonous. Therefore, results from our study are in keeping with the general observations in Roraima.

## Conclusions

As previously observed, results from this study demonstrate that the majority of malaria cases observed in the State of Roraima during our study period (2016–2018) were imported mainly from Venezuela with a good number coming from Guyana. This highlights the challenges for malaria control in transborder regions such as Pacaraima and Boa Vista in Roraima and in the Guiana Shield as a whole and the need for proactive assessment of the determinants and social factors that promote malaria transmission such as migration of people due to economic activities. These factors need to be taken into consideration when developing malaria control interventions in this region. In addition, international border surveillance and collaborative efforts toward malaria control and prevention are critical.

## Methods

This study was part of a larger cross-sectional surveillance study aimed at characterizing the epidemiological pattern of malaria through the spatial analysis of parameters related to the disease. The study was approved by the Federal University of Roraima Ethical Committee (CAAE: 44055315.0.0000.5302). The Centers for Disease Control and Prevention (CDC) investigators were not directly involved with enrollment of study subjects nor had access to any personally identifiable information (CDC project determination # 2017-105). Written informed consent was obtained from all participants. The study was performed in accordance with all relative guidelines and regulations.

### Study sites

The study was carried out in various malaria health posts, in three municipalities in Roraima state: Boa Vista, Pacaraima and Rorainópolis (Fig. 1). The malaria health posts are utilized by symptomatic patients seeking a malaria diagnosis and treatment. Boa Vista is the capital, with an estimated population of 399,213 inhabitants. It is 231.5 km from Santa Elena do Uairén, Venezuela and 133.3 km from Lethem, Guyana. Pacaraima is located on the border with Venezuela, with an estimated population of 17,401, including indigenous peoples of three ethnicities (Makuxi, Taurepang and Wapixana). The municipality has intense movement of people due to commerce. Rorainópolis is located in the south of the state, 298 km from Boa Vista. It is the second largest municipality in the state, with an estimated population of 30,163 inhabitants. Both Boa vista and Pacaraima municipalities have a low risk of malaria transmission. In contrast, Rorainópolis is a malaria endemic area with high number of autochthonous cases.

### Patient enrollment and sample collection

Samples and survey data were collected continuously from March 2016 to September 2018 in Boa Vista and Pacaraima. In Rorainópolis samples were collected in a single survey in 2018. Patients were approached for their willingness to participate in the study as they exited the malaria testing post after they had a routine malaria diagnosis by microscopy. The participants were recruited if they were coming from the health post where they had come to seek a malaria diagnosis and if they consented to participate in the study. All malaria positive patients were treated with antimalarial drugs, according to the Brazilian national therapeutic guidelines for malaria. Written informed consent was obtained from all participants. Consented individuals were asked to complete a questionnaire which was used to collect demographic data and travel history. Information about the patient’s country of residence, occupation, where the patient had lived in the last 15 days, whether they had travelled out of Brazil in the last 30 days and their travel destination. A 10 ml venous blood sample was obtained from each participating individual from which thin and thick blood smears and dried blood spots (DBS; Whatman 903 Protein Saver Cards) were prepared.

### Molecular diagnosis by photo-induced electron transfer-polymerase chain reaction (PET-PCR)

All the collected DBS were sent to Malaria Branch laboratory, CDC for malaria parasite confirmation. DNA was isolated from the collected DBS using the QIAmp DNA Mini Kit (QIAGEN, Valencia, CA) following the manufacturer's recommendations. The extracted DNA was aliquoted and stored at −20 °C until used for PCR. All samples were tested for malaria infection using PET-PCR assay as previously described^26^. Samples were first screened for *Plasmodium* using a genus-specific assay. *Plasmodium* positive samples were subsequently tested for species identification using two species-specific duplex assays: *P. falciparum*/*P. ovale* and *P. malariae*/*P. vivax*. Primers used for the PET-PCR assay are shown in Table 3*.* PET-PCR reaction assay was conducted using the Stratagene Mx3000P thermocycler machine (Agilent Technologies, Santa Clara, CA, USA).

**Table 3:** PET-PCR primers utilized in this study.

Primer name	Primer sequence
Genus forward	GGC CTA ACA TGG CTA TGA CG
Genus reverse-FAM	AGG CGC ATA GCG CCT GGC TGC CTT CCT TAG ATG TGG TAG CT
*P. falciparum* forward	ACC CCT CGC CTG GTG TTT TT
*P. falciparum* reverse-HEX	AGG CGG ATA CCG CCT GGT CGG GCC CCA AAA ATA GGA A
*P. malariae* forward	AAG GCA GTA ACA CCA GCA GTA
*P. malariae* reverse-FAM	AGG CGC ATA GCG CCT GGT CCC ATG AAG TTA TAT TCC CGC TC
*P. ovale* forward-FAM	AGG CGC ATA GCG CCT GGC CAC AGA TAA GAA GTC TCA AGT ACG ATA TT
*P. ovale* reverse	TTG GAG CAC TTT TGT TTG CAA
*P. vivax* forward	ACT GAC ACT GAT GAT TTA GAA CCC ATT T
*P. vivax* reverse-HEX	AGG CGC ATA GCG CCT GGT GGA GAG ATC TTT CCA TCC TAA ACC T

### Malaria cases classification

Malaria cases were classified as imported or autochthonous depending on the likely location of parasite acquisition, as per the participant-provided responses on the completed questionnaire. In this study, imported malaria was defined as malaria infection acquired outside Brazil but diagnosed within the three study sites. This was determined by the reported individual travel history of staying in an endemic country outside Brazil during the last 15 days. Autochthonous cases were defined as malaria acquired in Brazil as evidenced by lack of travel out of Brazil in the last 30 days and no evidence of importation.

### Data management and analysis

Descriptive statistics such as percentages, mean, median, standard deviation, and range were reported. The statistical significance of differences in proportions between groups was assessed by the chi-square test, differences in means by t-test when data followed a normal distribution using GraphPad InStat, version 3 (GraphPad Software, San Diego, CA, USA). The map was created in Quantum GIS software (QGIS version 2.14.17), using shapefile layers from South America and the state of Roraima. The coordinates of the municipalities of Boa Vista, Pacaraima and Rorainopolis was added to the map in addition to pie charts built in the Microsoft Excel.

### Consent for publication

Authors gave their consent for publication.

## Data Availability

The datasets supporting the conclusions of this article are included within the article.

## References

[1] WHO. World Malaria Report. (World Health Organization, Geneva, 2020).

[2] Epidemiological Alert: Increase of malaria in the Americas. (Pan American Health Organization 2018).

[3] Grillet ME (2019). Venezuela's humanitarian crisis, resurgence of vector-borne diseases, and implications for spillover in the region. Lancet Infect. Dis..

[4] Daniels JP (2018). Increasing malaria in Venezuela threatens regional progress. Lancet Infect. Dis..

[5] Valle D, Lima JM (2014). Large-scale drivers of malaria and priority areas for prevention and control in the Brazilian Amazon region using a novel multi-pathogen geospatial model. Malar. J..

[6] World Malaria Report (2020). 2020.

[7] Vreden SG, Jitan JK, Bansie RD, Adhin MR (2013). Evidence of an increased incidence of day 3 parasitaemia in Suriname: an indicator of the emerging resistance of Plasmodium falciparum to artemether. Mem. Inst. Oswaldo Cruz..

[8] SIVEP. http://200.214.130.44/sivep_malaria/.

[9] Louzada J (2020). The impact of imported malaria by gold miners in Roraima: characterizing the spatial dynamics of autochthonous and imported malaria in an urban region of Boa Vista. Mem. Inst. Oswaldo Cruz.

[10] Musset L (2014). Malaria on the Guiana Shield: A review of the situation in French Guiana. Mem. Inst. Oswaldo Cruz..

[11] Arisco NJ, Peterka C, Castro MC (2021). Cross-border malaria in Northern Brazil. Malar. J..

[12] de Pina-Costa A (2014). Malaria in Brazil: What happens outside the Amazonian endemic region. Mem. Inst. Oswaldo Cruz.

[13] Jaramillo-Ochoa R (2019). Effects of political instability in Venezuela on malaria resurgence at Ecuador-Peru Border, 2018. Emerg. Infect. Dis..

[14] Recht J (2017). Malaria in Brazil, Colombia, Peru and Venezuela: current challenges in malaria control and elimination. Malar. J..

[15] Oliveira-Ferreira J (2010). Malaria in Brazil: An overview. Malar. J..

[16] Siqueira AM (2016). Plasmodium vivax landscape in Brazil: scenario and challenges. Am. J. Trop. Med. Hyg..

[17] Castellanos A (2016). Malaria in gold-mining areas in Colombia. Mem. Inst. Oswaldo Cruz..

[18] da Silva-Nunes M (2012). Amazonian malaria: Asymptomatic human reservoirs, diagnostic challenges, environmentally driven changes in mosquito vector populations, and the mandate for sustainable control strategies. Acta Trop..

[19] Mosnier E (2020). Resurgence risk for malaria, and the characterization of a recent outbreak in an Amazonian border area between French Guiana and Brazil. BMC Infect. Dis..

[20] Chenet SM (2016). Independent emergence of the Plasmodium falciparum Kelch Propeller domain mutant allele C580Y in Guyana. J. Infect. Dis..

[21] Mathieu LC (2020). Local emergence in Amazonia of Plasmodium falciparum k13 C580Y mutants associated with in vitro artemisinin resistance. Elife.

[22] Pommier de Santi V (2016). Malaria Hyperendemicity and Risk for Artemisinin Resistance among Illegal Gold Miners, French Guiana. Emerg. Infect. Dis..

[23] Douine M (2018). Predictors of antimalarial self-medication in illegal gold miners in French Guiana: A pathway towards artemisinin resistance. J. Antimicrob. Chemother..

[24] Silva-Vasconcelos A, K. M., Mourao EN, de Souza RT, Lacerda RN, Sibajev A, Tsouris P, Póvoa MM, Momen H, Rosa-Freitas MG Biting indices, host-seeking activity and natural infection rates of anopheline species in Boa Vista, Roraima, Brazil from 1996 to 1998. *Mem Inst Oswaldo Cruz* **97**, 151–161 (2002). 10.1590/s0074-02762002000200002 12016435

[25] Tadei WP (1998). Ecologic observations on anopheline vectors of malaria in the Brazilian Amazon. Am. J. Trop. Med. Hyg..

[26] Lucchi NW (2013). Molecular diagnosis of malaria by photo-induced electron transfer fluorogenic primers: PET-PCR. PLoS ONE.

